# Pathologists’ Fees

**Published:** 1885-03

**Authors:** 


PATHOLOGISTS' FEES.

"Gentlemen! No money can be made out of dead men," said a distinguished pathological anatomist, a few months ago.

The remark was made to ten or twelve *internes* of one of our large metropolitan hospitals, who were collected around a dissecting table, noting with keenest interest, the deft strokes of the knife in the master's hand, and listening to his lucid interpretations of morbid processes. It was suggested by a word from one of the gentlemen, who expressed his intention of qualifying himself for similar activity.

The *animus* of the remark is obvious. The pathologist had learned, by bitter experience, the essential difference between liberal and professional education. He was anxious to deflect the attention of hot-blooded, impulsive youth,—inclined to reject the proposition that man "liveth by bread alone,"—from the contemplation of pure science, to what the Germans aptly term the *Brodwissenschaften*, which may be translated, *The Bread and Butter Sciences.*

In other words, the pathologist, in the selection of his figure, wished to impress upon the minds of his auditors an idea, old as the history of philosophy, admirably expressed by Sir Wm. Hamilton.

"It is manifest, indeed, that man, in so far as he is a mean for the glory of God, must be an end unto himself; for it is only in the accomplishment of his own perfection, that, as a creature, he can manifest the glory of his Creator. Though, therefore, man, by relation to God, be but a mean, for that very reason, in relation to all else is he an end."

But it is evident that under the condition of society, individual men are, for the most part, to a greater or less degree, actually diverted from the end of existence.

"To live, the individual must have the means of living; and these means (unless he already possess them) he must procure,—he must purchase. But purchase with what?

"With his services, *i. e.*—he must reduce himself to an instrument,—an instrument of utility to others; and the services of this instrument he must barter for those means of subsistence of which he is in want. In other words, he must exercise some trade, calling, or profession."

In America, pathology is as separate and distinct from *The Bread and Butter Sciences* as metaphysics.

Is this so? Up to the present time, this has undoubtedly been the case. We hope an era of better things is about to dawn.

The Chicago Gynecological Society, at its regular meeting, Dec. 19th, 1884, established an important precedent.

When Professor Byford's two specimens of "Mural Pregnancy" were referred to a pathological anatomist for examination, a motion, relative to *adequate* pecuniary compensation, was carried.

---

The recent occurrence of four destructive fires in insane hospitals in Indiana, Pennsylvania and Illinois, and the merely cursory interest manifested by the public press, indicates the necessity for organized effort to secure public attention to the interests of the insane. The Coroner's investigation of the origin of the fire at Kankakee found that the Medical Superintendent and the employès did all in their power to save life and property, but were hampered in their efforts by reason of lack of suitable alarm, fire escapes, and appliances for extinguishing fire. The medical superintendents in all the hospitals in which fire occurred were not only acquitted of negligence but commended for their zealous efforts to remove the inmates. In each case the fire was found to have originated in causes which the utmost vigilance has always failed to fully control. Defective construction of buildings, faulty heating apparatus and incendiarism are the common causes of such disasters and will continue to be despite the precautions prompted by personal interest or devotion to public duty. For obvious reasons, hospitals should be fire proof. The great loss of life in the Kankakee fire, despite the highly creditable efforts of Dr. Dewey and his assistants, and the difficulty and danger attending the rescue of incarcerated inmates of the Philadelphia Almshouse, ought to convince the public of this fact.

The rapid increase of insanity in this country is a fact so tardily recognized by our legislators, that provision for the safety and intelligent treatment of this unfortunate class rarely meets the requirements of common benevolence, and is yet more rarely adequate to the reasonable demands of professional management. The proportion of the insane to the population of California in 1860 was 1 to 883, in 1880, 1 to 383. In an article in this journal, November, 1883, Dr. Clevinger estimated the proportion of insane to the population of Chicago as 1 to 383. In our actively moving and rapidly increasing population, with a steady influx of European paupers, the causes will probably long continue to maintain if not to increase these proportions.

If the care of the insane has been assumed as a public duty, it is a crime to neglect the details essential to its performance. The Kankakee horror emphasizes this fact and suggests a searching inquiry into the general management of insane hospitals.

Such an inquiry would disclose the fact that the neglect to provide fire apparatus and a water supply for a building crowded with helpless inmates, is a fair illustration of legislative negligence. It would show that political influence, the collusions of privileged contractors, and the biennial junketings of legislative committees are not inspired by a benevolent interest in the welfare of the wards of the State. It would show that large sums are appropriated for architectural display, which, prudently expended, would afford security against fire. It would show that asylums are overcrowded, medical superintendents are overworked and the efficiency of their efforts abridged by factious opposition and political favoritism; that curable cases of mental disease are frequently neglected or placed under the care of unqualified physicians in charge of almshouses. A searching investigation would benefit the insane, the welfare of general society and the interests of medical science; it would develop an active and enlightened public interest that would inspire philanthropic effort and stimulate scientific progress. The medical profession should unite to promote this interest; the duty has grown with our knowledge of mental derangements and the essential for successful treatment, it has grown with a class of persistent and enthusiastic specialists who have labored under disadvantages which stupid opposition has unnecessarily increased, who have accomplished much for society and shared but few of its plaudits or substantial rewards. We are happy to observe increasing interests in the profession in this direction. The National Association for the Protection of the Insane is rapidly enlisting an influential membership who are urging clinical psychiatry in our medical colleges. Medical journals are insisting upon authoritative professional control of insane hospitals. The laity are beginning to realize the fact that insanity is a manifestation of physical disease in some degree amenable to medical treatment, that hospitals are provided, not merely for the custody of the insane to insure the safety of society, but chiefly for the cure and amelioration of disease. The correlative fact will soon be admitted, that the medical superintendent should be invested with the power to summarily discharge insubordinate employès, suspend assistants, in short, with the control of every detail of management which can in any manner influence the welfare of his irresponsible patients. The tenure of office should depend only upon the skill and fidelity of service. Under such conditions, if uniformly adopted, it would be possible to compare results, and fix the responsibility for failures; a sense of security would promote a degree of efficiency which can never exist while official positions in insane hospitals are the footballs of political acrobats.

